# Lumbricus Extract Prevents LPS-Induced Inflammatory Activation of BV2 Microglia and Glutamate-Induced Hippocampal HT22 Cell Death by Suppressing MAPK/NF-κB/NLRP3 Signaling and Oxidative Stress

**DOI:** 10.3390/cimb45120620

**Published:** 2023-12-11

**Authors:** You-Chang Oh, Yun Hee Jeong, Hye Jin Yang, Wei Li, Jin Yeul Ma

**Affiliations:** Korean Medicine (KM)-Application Center, Korea Institute of Oriental Medicine, 70, Cheomdanro, Dong-gu, Daegu 41062, Republic of Korea; runxi0333@kiom.re.kr (Y.H.J.); hjyang@kiom.re.kr (H.J.Y.); liwei1986@kiom.re.kr (W.L.)

**Keywords:** Lumbricus, anti-neuroinflammation, neuroprotective effects, antioxidant, anti-apoptosis

## Abstract

Microglia-induced inflammatory signaling and neuronal oxidative stress are mutually reinforcing processes central to the pathogenesis of neurodegenerative diseases. Recent studies have shown that extracts of dried *Pheretima aspergillum* (Lumbricus) can inhibit tissue fibrosis, mitochondrial damage, and asthma. However, the effects of Lumbricus extracts on neuroinflammation and neuronal damage have not been previously studied. Therefore, to evaluate the therapeutic potential of Lumbricus extract for neurodegenerative diseases, the current study assessed the extract’s anti-inflammatory and antioxidant activities in BV2 microglial cultures stimulated with lipopolysaccharide (LPS) along with its neuroprotective efficacy in mouse hippocampal HT22 cell cultures treated with excess glutamate. Lumbricus extract dose-dependently inhibited the LPS-induced production of multiple proinflammatory cytokines (tumor necrosis factor-α, interleukin (IL)-6, and IL-1β) and reversed the upregulation of proinflammatory enzymes (inducible nitric oxide synthase and cyclooxygenase-2). Lumbricus also activated the antioxidative nuclear factor erythroid 2-relayed factor 2/heme oxygenase-1 pathway and inhibited LPS-induced activation of the nuclear factor-κB/mitogen-activated protein kinases/NOD-like receptor family pyrin domain containing 3 inflammatory pathway. In addition, Lumbricus extract suppressed the glutamate-induced necrotic and apoptotic death of HT22 cells, effects associated with upregulated expression of antiapoptotic proteins, downregulation of pro-apoptotic proteins, and reduced accumulation of reactive oxygen species. Chromatography revealed that the Lumbricus extract contained uracil, hypoxanthine, uridine, xanthine, adenosine, inosine, and guanosine. Its effects against microglial activation and excitotoxic neuronal death reported herein support the therapeutic potential of Lumbricus for neurodegenerative diseases.

## 1. Introduction

Uncontrolled neuroinflammation and oxidative stress contribute to the pathogenesis of neurodegenerative diseases [1]. Microglia are immune cells that reside within the central nervous system and are the primary initiators of neuroinflammatory signaling in response to infections and neuronal damage of various etiologies. Persistent activation of microglia by stimuli such as the bacterial toxin lipopolysaccharide (LPS) and damage-associated molecular patterns (DAMPs) from injured neurons induces the release of inflammatory mediators including tumor necrosis factor (TNF)-α, interleukin (IL)-6, IL-1β and nitric oxide (NO). These factors can in turn exacerbate both neuroinflammation and neuronal injury, creating a mutually reinforcing pathogenic process that is strongly implicated in the progression of both acute brain damage and various neurodegenerative disorders [2]. Oxidative stress is characterized by an imbalance between reactive oxygen species (ROS) production and cellular antioxidant capacity, and also contributes to neurodegeneration by damaging cellular macromolecules (such as genomic DNA, membrane lipids, and various structural and function proteins), ultimately leading to necrotic and apoptotic cell death [3,4]. Further, excessive inflammatory responses in the brain increase ROS production, while oxidative stress triggers the inflammatory activation of microglia. Therefore, the inflammatory response and oxidative stress may interact to exacerbate neurodegeneration [5].

The mitogen-activated protein kinases (MAPKs) including extracellular signal-regulated kinase (ERK), p38, and c-Jun NH_2_-terminal kinase (JNK) [6] and the transcription factor nuclear factor (NF)-κB are major signaling factors controlling microglial activation and neuroinflammation [7]. Activation of MAPKs through phosphorylation by various upstream signaling pathways stimulates the transcriptional activity of NF-κB via phosphorylation of the inhibitory subunit [7], after which the NF-κB p65 subunit translocates to the nucleus and promotes the production of inflammatory mediators in microglia including TNF-α, IL-6, and IL-1β [8]. Therefore, targeting the MAPK/NF-κB pathways may be an effective therapeutic approach for the prevention and treatment of neuroinflammatory diseases.

Conversely, the stress responsive protein heme oxygenase (HO)-1 is known to reduce the secretion of proinflammatory mediators such as NO and various cytokines [9]. Expression of HO-1 is induced by activation of the redox-dependent transcription factor nuclear factor erythroid 2-relayed factor 2 (Nrf-2) during inflammatory processes through translocation to the nucleus, and recent studies have confirmed the importance of this Nrf-2/HO-1 signaling pathway for controlling the inflammatory activity of macrophages and microglia [10,11].

Initiation of the inflammatory signaling cascade is dependent on the formation of a multiprotein inflammasome including the NOD-like receptor family pyrin domain containing 3 (NLRP3). This NLRP3 complex first activates the protease pro-caspase-1 [12], which then cleaves the IL-1β precursor pro-IL-1β to produce mature active IL-1β [12]. Activation of NLRP3 and ensuing generation of IL-1β and IL-18 can induce mitochondrial dysfunction and ROS accumulation, resulting in neurodegeneration [13]. Stimulation of the P2X purinoceptor 7 (P2X7) by ATP release from damaged cells or by *E. coli* bacterial endotoxin also activates NLRP3 in monocytes/macrophages [14], while Sirtuin 2 (SIRT2), a class III nicotinamide adenine dinucleotide-dependent deacetylase ubiquitously expressed in cellular nuclei and cytoplasm [15], can prevent acetylation-dependent activation of the NLRP3 inflammasome under conditions such as aging and nutrient overload [16,17]. Targeted regulation of NLRP3 activity is thus considered another promising strategy for inhibiting neuroinflammation and associated ROS accumulation and excessive autophagy, thereby slowing the progression of neurodegenerative diseases [18].

Neurotransmitter imbalance is also a major cause of synaptic loss and cell death in acute and chronic neurodegenerative diseases [19]. For instance, excessive extracellular accumulation of the excitatory neurotransmitter glutamate and concomitant overstimulation of neuronal glutamate receptors triggers a cascade of pathogenic processes, including calcium overload, mitochondrial dysfunction, and oxidative stress caused by excessive accumulation of ROS (collectively termed excitotoxicity), that contributes to neurodegenerative disorders such as Alzheimer’s disease [4,20]. Therefore, suppression of excessive ROS production is also essential for the prevention of neurodegenerative diseases.

*Pheretima aspergillum* (Megascolecidae) and various derivative products have long been used in traditional oriental medicine to treat high fever, seizures in children, asthma, and high blood pressure. Previous studies have demonstrated that the dried bodies of *P. aspergillum* (termed Lumbricus) reduce fibrosis, inhibit mitochondrial damage, and relieve asthma [21,22,23]. However, the efficacies of Lumbricus extracts against microglia-induced neuroinflammation and hippocampal cell excitotoxicity have not been evaluated. Therefore, in this study, we investigated the anti-neuroinflammatory activity of Lumbricus extracts on LPS-stimulated BV2 microglia and the neuroprotective effects on glutamate-stimulated HT22 hippocampal cells. In addition, both the underlying molecular mechanisms and the chemical compositions of Lumbricus extracts were investigated to establish relationships between bioactive ingredients and therapeutic effects.

## 2. Materials and Methods

### 2.1. Materials and Reagents

Dulbecco’s modified Eagle’s medium (DMEM), penicillin/streptomycin antibiotics, and fetal bovine serum (FBS) were obtained from Hyclone (Logan, UT, USA). All cell culture dishes and plates were acquired from SPL Life Sciences (Pocheon, Republic of Korea). LPS, glutamate, bovine serum albumin (BSA), and dimethyl sulfoxide (DMSO) were obtained from Sigma–Aldrich (St. Louis, MO, USA). A Cell Counting Kit (CCK; CK04) was acquired from Dojindo Molecular Technologies (Kumamoto, Japan), Griess reagent for nitrite detection from Sigma–Aldrich, and enzyme-linked immunosorbent assay (ELISA) kits (TNF-α: 88-7324-77, IL-6: 88-7064-77) from Invitrogen (Carlsbad, CA, USA). An RNA extraction kit (17061) was purchased from iNtRON Biotech (Daejeon, Republic of Korea), while oligonucleotide primers for real-time reverse transcription-polymerase chain reaction (RT-qPCR), cDNA synthesizing kits (K-2044-B), and AccuPower^®^ 2x GreenStar qPCR Master Mix (K-6251) were purchased from Bioneer (Daejeon, Republic of Korea). Various primary antibodies and horseradish peroxidase (HRP)-conjugated secondary antibodies for Western blot analysis were acquired from Cell Signaling Technology (Danvers, MA, USA). Polyvinylidene difluoride (PVDF) membranes were obtained from Millipore (Bedford, MA, USA). A lactate dehydrogenase (LDH) assay kit (ab102526) was purchased from Abcam, Inc. (Cambridge, UK) and 2′,7′-dichlorofluorescein diacetate (H_2_DCFDA; D399) from Invitrogen. A Lipid Peroxidation Assay kit (ab118970) for determination of malondialdehyde (MDA) was acquired from Abcam, Inc. and an Annexin V-FITC/propidium iodide (PI) apoptosis detection kit (556547) from BD Biosciences (Franklin Lakes, NJ, USA). High-performance liquid chromatography (HPLC) analysis was performed using a Dionex UltiMate 3000 system (Dionex Corp., Sunnyvale, CA, USA) equipped with a binary pump, auto-sampler, column oven, and diode array UV/VIS detector (DAD). System control and data analysis were achieved using Dionex Chromelon software.

### 2.2. Preparation of Lumbricus Water Extract (LWE) and Lumbricus Ethanol Extract (LEE)

Lumbricus was purchased from Yeongcheonhyundai Herbal Market (Yeongcheon, Republic of Korea) and authenticated by Prof. KiHwan Bae of the College of Pharmacy, Chungnam National University (Daejeon, Republic of Korea). All voucher specimens were deposited in an herbal bank at the Korean Medicine-Application Center, Korea Institute of Oriental Medicine (voucher number: W201 and E201). For preparation of LWE, 50.0 g of Lumbricus was boiled in 1 L of water for 3 h. For preparation of LEE, 50.0 g of Lumbricus was incubated in 300 mL of 70% ethanol at 40 °C in a shaking incubator (100 rpm) for 24 h. Extract solutions were filtered through 150-mm filter paper (Whatman, Piscataway, NJ, USA) and concentrated using a rotary vacuum evaporator (Buchi, Tokyo, Japan). Samples were then freeze-dried and kept in desiccators at −20 °C until use.

### 2.3. Cell Culture and Drug Treatment

The murine microglial cell line BV2 were obtained from Professor Kyoungho Suk at Kyungpook National University (Daegu, Republic of Korea) and grown in DMEM supplemented with 10% FBS and 1% penicillin/streptomycin antibiotics. The hippocampal HT22 cells were obtained from Dr. Younghoon Go at Korea Institute of Oriental Medicine (Daegu, Republic of Korea) and grown in complete DMEM. Cultures were maintained in a humidified incubator at 37 °C under a humidified 95% air/5% CO_2_ atmosphere. The culture medium was exchanged every 2 days, and cells were grown to 80–90% confluence for experiments. The cells were treated with 100 ng/mL LPS or 5 mM glutamate in the presence or absence of LWE or LEE (10–300 μg/mL). For cellular applications, LPS and glutamate were dissolved in distilled water (DW). For application to cells, LWE and LEE lyophilized powder was dissolved using 50% DMSO (50% DW). The final DMSO concentration of each LWE and LEE sample applied to cells was 0.005–0.15%.

### 2.4. Cell Viability

For experiments, BV2 cells were seeded at 1 × 10^4^ cells/well in 96-well plates and cultured for 18 h, while HT22 cells were seeded at 5 × 10^3^ cells/well and cultured for 24 h before the indicated treatment. To estimate viable cell number, 10 μL of CCK solution was added to each well for 1 h and the absorbance read at 450 nm using a microplate reader (SpectraMax i3, Molecular Devices, San Jose, CA, USA). In addition, for cell counting using trypan blue (TB) staining, DMEM medium containing cells and TB were diluted in the same ratio. Each stained cell was counted using the LUNA-II™ Automated Cell Counter (Logos Biosystems, Inc., Anyang, Republic of Korea). The proportion of viable cells relative to vehicle-treated controls was calculated.

### 2.5. Analysis of NO Secretion

Nitric oxide secretion was estimated by measuring the nitrite concentration in the culture medium using Griess reagent. Briefly, BV2 cells were plated into 96-well plates at 5 × 10^4^ cells/well, preincubated with LWE for 1 h, and exposed to LPS for 24 h. Cells were then incubated in 100 μL/well Griess reagent at room temperature (RT) for 5 min, and absorbance was measured at 570 nm using a microplate reader.

### 2.6. ELISA for Cytokine Determination

Cytokine concentrations were measured in the culture medium using an ELISA kit according to the manufacturer’s protocol. Briefly, BV2 cells were seeded in 24-well plates at 2.5 × 10^5^ cells/well and incubated for 18 h. The cells were then pretreated with 100 or 300 μg/mL LWE for 1 h and further challenged with LPS for an additional 6 h. After incubation, proinflammatory cytokine levels were measured in the collected medium supernatant as previously reported [24].

### 2.7. Total RNA Extraction, DNA Synthesis, and RT-qPCR

Total RNA was extracted using the easy-BLUE™ RNA extraction kit (iNtRON Biotech) and reverse transcribed using AccuPower^®^ CycleScript RT PreMix (Bioneer). Detailed methods and conditions for RT-qPCR are referred to in our previous study [25]. Primer sequences are listed in Table 1 [25,26]. The following PCR conditions were applied: TNF-α, IL-6, IL-1β, iNOS, cyclooxygenase (COX)-2, HO-1, and β-actin, with 40 cycles of 94 °C for 15 s and 60 °C for 1 min. Amplification and analysis were performed using a QuantStudio 6 Flex Real-time PCR System (Thermo Fisher Scientific, Rockford, IL, USA). Relative gene expression was calculated using the relative CT method and presented as fold-change, which was measured as the internal control.

### 2.8. Preparation of Whole-Cell, Cytosolic, and Nuclear Lysates

To obtain whole-cell lysate fractions, cells were treated as indicated, harvested, centrifuged, and resuspended in radioimmunoprecipitation assay lysis buffer (Millipore) containing protease and phosphatase inhibitor cocktail (Roche, Basel, Switzerland). Cytosolic and nuclear fractions were isolated using NE-PER™ nuclear and cytoplasmic extraction reagents (Thermo Fisher Scientific; 78833) according to the manufacturer’s protocol. The fractions were stored at −80 °C until analysis.

### 2.9. Western Blot Analysis

Total lysate protein concentrations were first measured by Bradford assay [27]. An amount of 20 μg of total protein were then separated by sodium dodecyl sulfate-polyacrylamide gel electrophoresis and transferred onto PVDF membranes overnight. The membranes were blocked with 3% BSA at RT for 1 h, incubated with the indicated primary antibodies overnight at 4 °C, washed four times with tris-buffered saline containing 0.1% Tween 20, then incubated with HRP-labeled species-specific secondary antibodies at RT for 1 h. Target protein bands were visualized using the ChemiDoc^TM^ Touch Imaging System (Bio-Rad, Hercules, CA, USA) and quantified using ImageJ (version 1.53k). Band densities were normalized to β-actin, TATA-box binding protein (TBP), or each total-type protein expression as the gel loading control. The primary and secondary antibodies employed are listed in Table 2.

### 2.10. LDH Assay for Cytotoxicity

Cytotoxicity was analyzed by measuring LDH released into the culture medium by dead cells. Briefly, HT22 cells were seeded at 5.0 × 10^3^/well in 96-well plates, preincubated with LEE for 1 h, and exposed to 5 mM glutamate for 24 h. The medium supernatant was obtained by centrifugation, and 10 μL per well transferred to new plates. The LDH reaction mix (100 μL/well) was added to each well, and incubated for an additional 30 min. LDH activity was calculated by measuring absorbance at 450 nm. Cytotoxicity was expressed as a percentage of LDH release relative to vehicle-treated control cells.

### 2.11. Intracellular ROS Determination

Changes in intracellular ROS were measured fluorometrically using H_2_DCFDA according to methods in a previous report [28]. Briefly, cells were treated as indicated with LEE and glutamate, washed twice in phosphate-buffered saline (PBS), and stained with 20 μM of H_2_DCFDA for 30 min at 37 °C in the dark. The stained cells were washed twice with PBS. A fluorescence microplate reader at an excitation wavelength of 488 nm and emission wavelength of 525 nm was used to analyze dihydroethidium fluorescence. Representative images were also obtained using a fluorescence microscope (Eclipse Ti, Nikon, Tokyo, Japan).

### 2.12. Measurement of MDA

A total of 2.0 × 10^6^ HT22 cells were treated with LEE and 5 mM glutamate, harvested 6 h later, and dissolved in 300 μL of MDA lysis buffer containing thiobarbituric acid. Samples were homogenized by vortex 6 times for 1 min per every 10 min and by sonication for 10 min and stored on ice. MDA concentration was assessed by the colorimetric method using the Lipid Peroxidation Assay kit according to the manufacturer’s instructions, and absorbance was measured at 532 nm using a microplate reader.

### 2.13. Apoptotic Cell Death Assessment by Flow Cytometry

Cellular apoptosis was assessed by flow cytometry using an FITC-Annexin V staining kit according to the manufacturer’s protocol. Cells were harvested after a 24 h exposure to glutamate in the presence or absence of LEE and stained with propidium iodide (PI) and FITC-conjugated Annexin V for 15 min at RT under darkness. Cell staining was quantified to measure early and late apoptosis using an FACS Calibur system (BD Biosciences).

### 2.14. Preparation of Extracts and Standards for HPLC

For HPLC analysis, LWE and LEE extract samples as well as seven standard compounds were dissolved in 50% methanol solution. The LWE and LEE concentrations were adjusted to 20 mg/mL, while standard stock solutions were initially prepared at 1.0 mg/mL and serially diluted in methanol to generate calibration curves spanning from 25 to 100 μg/mL. All solutions were passed through 0.45 μm RC membrane syringe filters prior to loading on the HPLC system (Sartorius, Germany).

### 2.15. Chromatographic Conditions

Chromatographic separation was conducted using a Luna C18 column (250 × 4.6 mm, 5 μm, Phenomenex, Torrance, CA, USA) maintained at 30 °C by a column oven, and eluents detected as 254 nm. The mobile phase consisted of 0.1% formic acid in water (solvent A) and acetonitrile (solvent B) flowing at 1 mL/min with the following gradient protocol: 0–10 min, 0.2% B; 10–25 min, 3% B; 25–30 min, 6.5% B; and 30–40 min, 100% B. To ensure proper equilibration before injection of the next sample, the column was re-equilibrated with the initial solvent (0.2% B) for 15 min.

### 2.16. Validation of the Method

Validation of the method involved assessing specificity by comparing the chromatographic profile of the standard with that of the sample extract. Linearity was evaluated by calculating the correlation coefficient (*R*^2^) of the calibration curve for each compound within a concentration range of 0.16 to 225.0 μg/mL. Regression equations were determined using the formula *y = ax ± b*, where y and x represent the peak areas and concentrations of the sample, respectively.

### 2.17. Statistical Analysis

All results are presented as mean ± standard error of the mean from three independent experiments. Treatment group means were compared by one-way analysis of variance followed by post-hoc Dunnett’s tests for comparing the LPS or glutamate sample with each extract-treated sample. A *p* < 0.05 was considered statistically significant for all tests. Statistical analyses were conducted using GraphPad Prism version 5.02 (GraphPad Software, Inc., San Diego, CA, USA).

## 3. Results

### 3.1. Effects of LWE on Microglial Cell Viability

**Figure 1:**
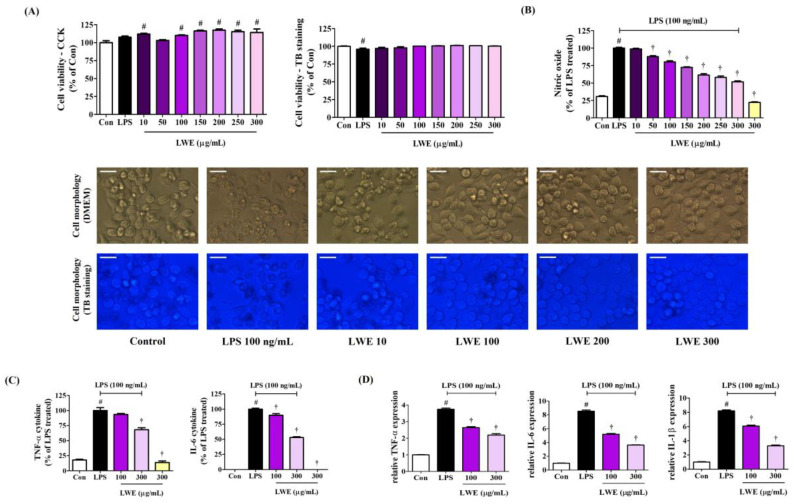
Effects of LWE on (**A**) viability and morphology, (**B**) secretion of NO, (**C**) production of inflammatory cytokine, and (**D**) cytokine mRNA expression in BV2 cells. (**A**) BV2 cells were incubated with 100 ng/mL of LPS or LWE concentrations of 10–300 μg/mL. (**B**–**D**) After LWE pretreatment, BV2 cells were stimulated with 100 ng/mL of LPS. (**A**) The images represent the three independent experiments. Scale bar = 10 μm. (**D**) RT-qPCR results are presented from six independent experiments. Control cells were incubated with the vehicle alone. Data are presented as mean ± standard error of the mean. # *p* < 0.05 vs. control; † *p* < 0.001 vs. LPS.

We first examined the influence of LWE on the viability of BV2 mouse microglia using the CCK solution. Exposure to LWE concentrations up to 300 μg/mL for 24 h did not reduce viable cell number compared to untreated control cultures (Figure 1A). Moreover, we cross-validated cell viability through TB staining to reliably rule out the potential cytotoxicity of LWE, and as with the CCK results, viability was similar to that of the control cells at all concentrations (Figure 1A). In addition, when observing cells in the culture medium and cells stained with TB, treatment with LWE had no significant effect on the morphology of BV2 cells at any concentration (Figure 1A). Thus, LWE concentrations from 10 to 300 μg/mL were examined for anti-neuroinflammation activities. Additionally, when treated with 100 ng/mL LPS alone, the survival rate of BV2 microglial cells changed only slightly, but the morphology of the cell line observed through microscope showed a significant change due to stimulation by LPS.

### 3.2. LWE Dose-Dependently Inhibited the Secretion of NO and the Production of Inflammatory Cytokines by LPS-Stimulated BV2 Microglia

To investigate the anti-neuroinflammatory efficacy of LWE, we first evaluated effects on the secretion of NO following LPS stimulation using the Griess assay, which detects nitrite derivates in the culture medium. As expected, LPS treatment alone strongly elevated extracellular nitrite levels, consistent with enhanced NO release, whereas pretreatment with LWE dose-dependently diminished extracellular nitrite accumulation following LPS exposure (Figure 1B), suggesting reduced LPS-stimulated NO production. Further, LWE treatment dose-dependently suppressed LPS-induced proinflammatory cytokine release and mRNA expression as evidenced by ELISA and RT-qPCR, respectively (Figure 1C,D). In addition, when the highest concentration of 300 μg/mL LWE was treated alone, NO and cytokine release did not increase compared to control cells, meaning that LWE itself does not cause an inflammatory response in microglial cells.

### 3.3. LWE Reduced MAPK Phosphorylation and NF-κB Nuclear Translocation in LPS-Stimulated BV2 Cells

**Figure 2:**
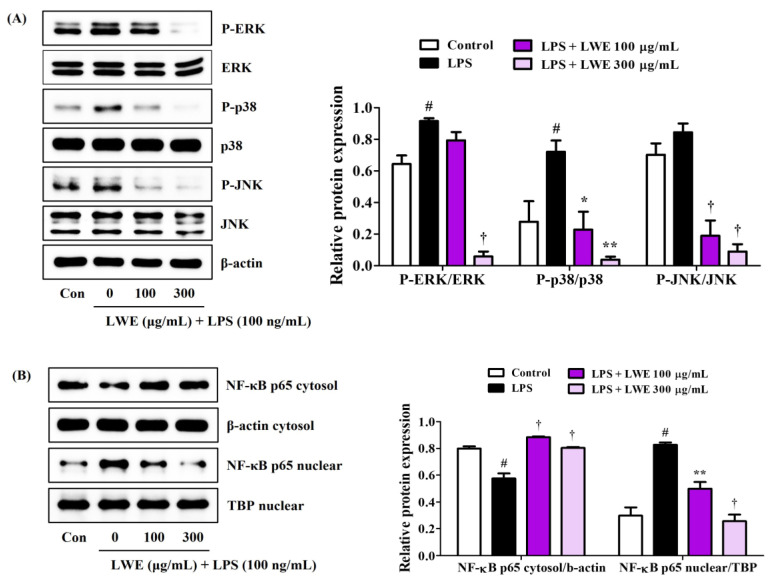
Effects of LWE on (**A**) the phosphorylation of MAPK and (**B**) the nuclear translocation of NF-κB p65. After LWE pretreatment at concentrations of 100 or 300 μg/mL, BV2 cells were stimulated with 100 ng/mL of LPS. Control cells were incubated with the vehicle alone. Blot images represent three independent experiments. Data are presented as mean ± standard error of the mean. # *p* < 0.05 vs. control; * *p* < 0.05, ** *p* < 0.01, and † *p* < 0.001 vs. LPS.

The phosphorylation of MAPK by upstream inflammatory inducers (such as bacterial toxins and DAMPs) and the ensuing activation of NF-κB transcriptional activity are required for the upregulation and release of many proinflammatory factors, so we examined the influence of LWE on MAPK and NF-κB pathway activities in LPS-stimulated BV2 microglial cells. Western blot analysis revealed that LWE dose-dependently inhibited phosphorylation of the MAPKs ERK, p38, and JNK (Figure 2A) and also strongly reduced translocation of the NF-κB active subunit p65 from cytoplasm to nucleus (Figure 2B).

### 3.4. LWE Pretreatment Suppressed LPS-Induced Expression of iNOS and COX-2

**Figure 3:**
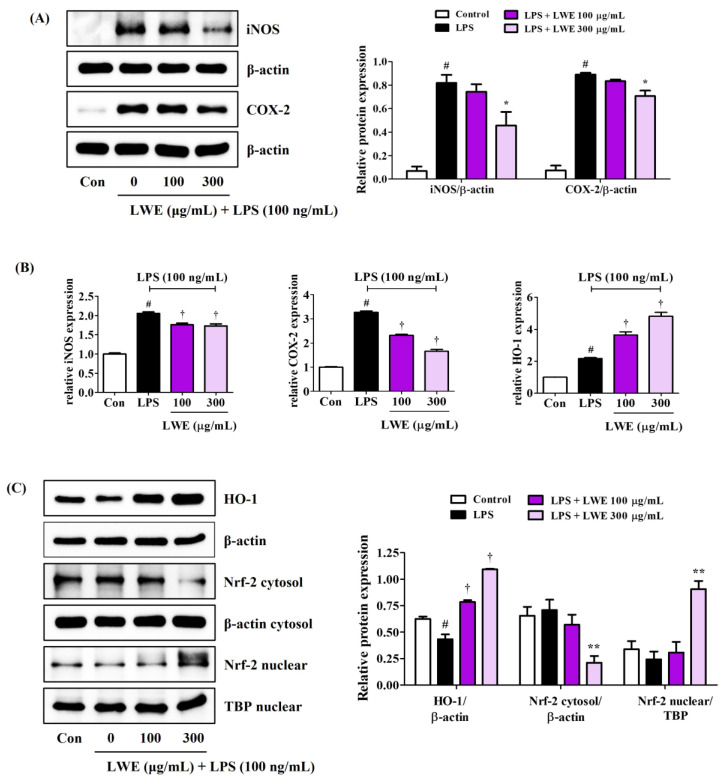
Effects of LWE on (**A**) the protein expression of iNOS and COX-2, (**B**) the mRNA expression of iNOS, COX-2, and HO-1, and (**C**) the nuclear translocation of Nrf-2 and protein expression of HO-1. After LWE pretreatment at concentrations of 100 or 300 μg/mL, BV2 cells were stimulated with 100 ng/mL of LPS. (**B**) RT-qPCR results are presented from six independent experiments. Control cells were incubated with the vehicle alone. Blot images represent three independent experiments. Data are presented as mean ± standard error of the mean. # *p* < 0.05 vs. control; * *p* < 0.05, ** *p* < 0.01, and † *p* < 0.001 vs. LPS.

Consistent with reduced NO production, LWE reduced LPS-stimulated expression of iNOS at both protein and mRNA levels (Figure 3A,B). In addition, LWE pretreatment inhibited LPS-induced upregulation of COX-2 protein and mRNA (Figure 3A,B), suggesting reduced production of proinflammatory prostaglandins.

### 3.5. LWE Upregulated HO-1 and Nuclear Translocation of Nrf-2 in LPS-Stimulated BV2 Microglia

In addition to suppressing LPS-induced upregulation of proinflammatory cytokines, iNOS, and COX-2, pretreatment with 300 μg/mL LWE also strongly upregulated antioxidant HO-1 protein expression, while concentrations ≥100 μg/mL also upregulated HO-1 mRNA expression (Figure 3B,C). Further, 300 μg/mL LWE enhanced the nuclear translocation of Nrf-2, which is required for HO-1 induction (Figure 3C).

### 3.6. LWE Inhibited LPS-Induced Expression of NLRP3 Inflammasome-Related Proteins in BV2 Cells

**Figure 4:**
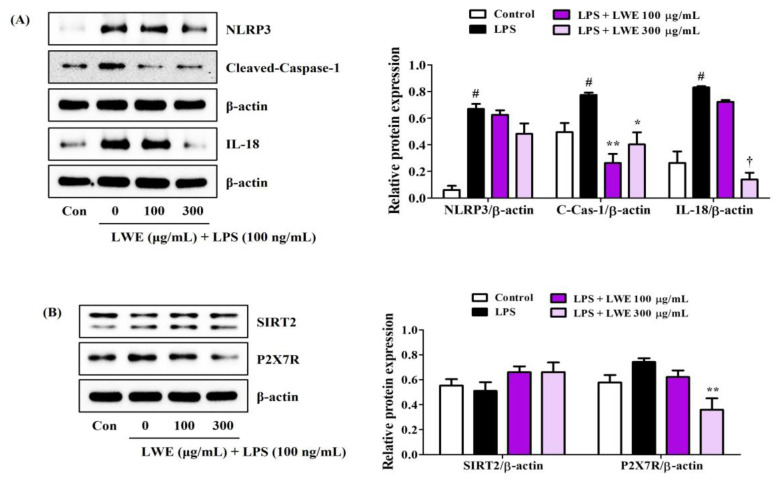
Effects of LWE on the expression of (**A**) NLRP3 inflammasome constituent proteins and (**B**) NLRP3 activity regulatory proteins. After LWE pretreatment at concentrations of 100 or 300 μg/mL, BV2 cells were stimulated with 100 ng/mL of LPS. Control cells were incubated with the vehicle alone. Blot images represent three independent experiments. Data are presented as mean ± standard error of the mean. # *p* < 0.05 vs. control; * *p* < 0.05, ** *p* < 0.01, and † *p* < 0.001 vs. LPS.

We next investigated whether the anti-neuroinflammatory activity of LWE is associated with inhibition of the NLRP3 inflammasome. Indeed, Western blot analysis revealed that LWE effectively inhibited LPS-induced upregulation of NLRP3, cleaved-caspase-1, and IL-18 proteins (Figure 4A). In addition, SIRT2, an enzyme known to inhibit NLRP3 activity, was very slightly reduced by LPS treatment and slightly recovered by LWE pretreatment, but no significant changes were observed (Figure 4B). Pretreatment with LWE also reversed the LPS-induced upregulation of P2X7, a known promoter of NLRP3 activation (Figure 4B).

### 3.7. LEE Protected Hippocampal HT22 Cells against Glutamate-Induced Death

**Figure 5:**
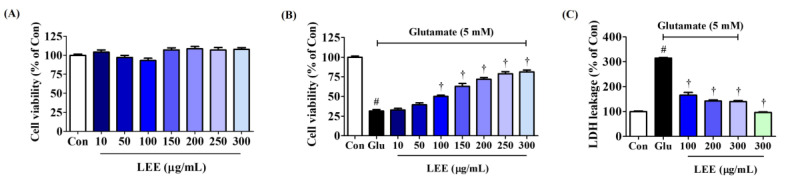
Effects of LEE on glutamate-induced cytotoxicity in HT22 cells. (**A**) HT22 cells were incubated with LEE concentrations of 10–300 μg/mL. (**B**,**C**) After LEE pretreatment at concentrations of 10–300 μg/mL, HT22 cells were exposed to 5 mM of glutamate. Control cells were incubated with the vehicle alone. Data are presented as mean ± standard error of the mean. # *p* < 0.05 vs. control; † *p* < 0.001 vs. glutamate.

To evaluate the direct neuroprotective efficacy of Lumbricus, we assessed the effects of LEE on glutamate-induced neurotoxicity (excitotoxicity) in cultures of murine hippocampal HT22 cells. Pretreatment with LEE had no influence on cell viability at concentrations up to 300 μg/mL (Figure 5A), but dose-dependently reversed glutamate-induced excitotoxicity by up to 80% (Figure 5B). Further, LEE pretreatment significantly reduced glutamate-induced LDH leakage (Figure 5C), confirming mitigation of glutamate-induced cell death. Additionally, treatment with 300 μg/mL LEE alone did not increase LDH leakage compared to control cells, indicating that LEE itself did not cause neurotoxicity to hippocampal cells.

### 3.8. LEE Reduced Glutamate-Induced Intracellular ROS Generation and MDA Expression in HT22 Cells

**Figure 6:**
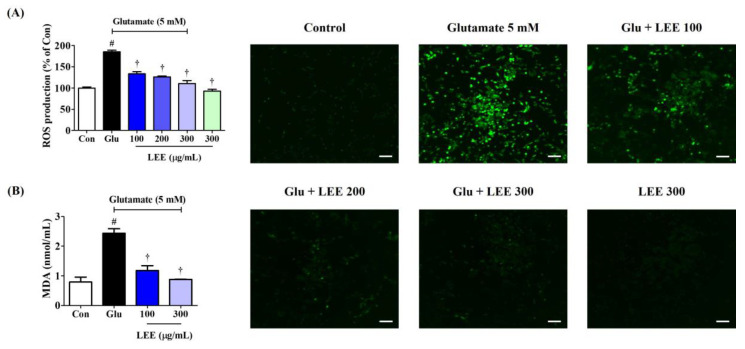
Effects of LEE against glutamate-induced (**A**) intracellular ROS production and (**B**) MDA expression in HT22 cells. Cells were pretreated with LEE concentrations of 10–300 μg/mL and then with 5 mM of glutamate. (**A**) H_2_DCFDA (20 μM) is an oxidation-sensitive fluorescence dye used to assess ROS levels. The ROS production was determined using a fluorescence microplate reader and a fluorescence microscope. Scale bar = 200 μm. (**B**) Levels of MDA were measured using a Lipid Peroxidation Assay kit. Control cells were incubated with the vehicle alone. All experiments were repeated at least three times, and similar results were obtained. Data are presented as mean ± standard error of the mean. # *p* < 0.05 vs. control; † *p* < 0.001 vs. glutamate.

Excitotoxicity is associated with oxidative stress, so we examined if LEE (10, 100, 200, 250, and 300 μg/mL) could reduce ROS accumulation in glutamate-treated HT22 cells using the ROS-sensitive fluorescent dye H_2_DCFDA and fluorescence spectroscopy. Pretreatment with LEE dose-dependently reduced glutamate-induced ROS generation as indicated by lower peak fluorescence emission (Figure 6A). Moreover, qualitatively similarly effects were found under fluorescence microscopy. Additionally, as with LDH, ROS production did not increase when the samples were treated with 300 μg/mL LEE alone, meaning that LEE itself does not cause oxidative stress. MDA expression, which indicates the level of lipid peroxidation caused by oxidative stress, also showed a significant increase by glutamate treatment and was suppressed in a concentration-dependent manner by LEE treatment (Figure 6B).

### 3.9. LEE Suppressed Glutamate-Induced Apoptosis of HT22 Cells

**Figure 7:**
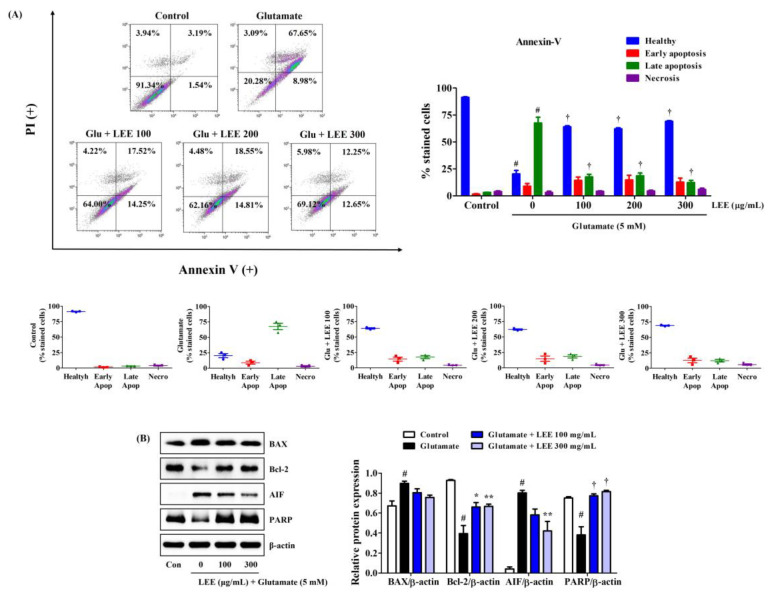
Effects of LEE on glutamate-induced apoptosis in HT22 cells. Cells were pretreated with LEE at concentrations of 100–300 μg/mL and then treated with 5 mM glutamate. (**A**) Apoptosis of HT22 cells was evaluated using flow cytometry. The image on the top right shows the percentage of healthy, early apoptotic, late apoptotic, and necrotic cells for each treatment group. The dot plot images in the middle represents the percentage of healthy, early apoptotic, late apoptotic, and necrotic cells for each treatment group in three independent experiments. (**B**) The expression levels of BAX, Bcl-2, AIF, and PARP were determined by Western blot analysis. Control cells were incubated with the vehicle alone. All experiments were repeated at least three times, and similar results were obtained. Data are presented as mean ± standard error of the mean. # *p* < 0.05 vs. control; * *p* < 0.05, ** *p* < 0.01, and † *p* < 0.001 vs. glutamate.

Consistent with CCK and LDH assays, LEE pretreatment (100, 200, or 300 μg/mL) markedly reduced glutamate-induced HT22 cell apoptosis as measured by Annexin V/PI staining and flow cytometry (Figure 7A). Moreover, consistent with flow cytometry results, Western blotting revealed that LEE pretreatment reversed the glutamate-induced upregulation of apoptosis-inducing factor (AIF) as well as the glutamate-induced downregulation of antiapoptotic proteins B-cell lymphoma 2 (Bcl-2) and poly (ADP-ribose) polymerase (PARP) (Figure 7B). However, glutamate-induced expression of pro-apoptotic proteins Bcl-2-associated X (BAX) was slightly suppressed by LEE pretreatment and did not reach significance (Figure 7B).

### 3.10. Identification of Potential Bioactive Components in LWE and LEE Using HPLC-DAD

**Figure 8:**
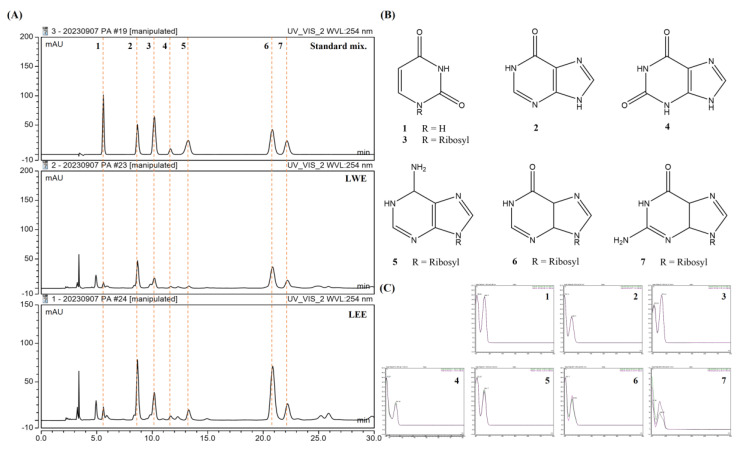
Potential bioactive components of LWE and LEE revealed by HPLC. (**A**,**B**) HPLC chromatograms (**A**) and structures of seven major compounds (**B**) detected in LWE and LEE. The detection wavelength was 254 nm. (**C**) Retention times for uracil (1), hypoxanthine (2), uridine (3), xanthine (4), adenosine (5), inosine (6), and guanosine (7).

We selected seven standard compounds to assess the compositions of these extracts using HPLC (Figure 8A), and subsequently detected all seven in both LWE and LEE samples based on retention times and UV spectra (Figure 8B,C): uracil (1), hypoxanthine (2), uridine (3), xanthine (4), adenosine (5), inosine (6), and guanosine (7).

### 3.11. Validation of the Analytical HPLC Method

The analytical HPLC method was validated by constructing calibration curves for seven major compounds. These curves were generated by plotting peak area against concentration, utilizing least-squares regression analysis. Each calibration equation was derived from five concentrations, spanning from 0.16 to 225.0 μg/mL. The high linear correlation coefficient (*R*^2^ > 0.99) for all calibration curves indicated a robust linear relationship. Based on these curves, the amounts of compounds 1 to 7 in the LWE extract were determined to be 0.15 (1), 0.99 (2), 1.11 (3), 0.03 (4), 0.17 (5), 3.18 (6), and 0.75 (7) mg/g, respectively. Similarly, the amounts of compounds 1 to 7 in the LEE extract were found to be 0.27 (1), 1.70 (2), 2.30 (3), 0.07 (4), 0.71 (5), 6.02 (6), and 1.34 (7) mg/g, respectively (Table 3).

## 4. Discussion

Substances with anti-inflammatory and antioxidant efficacy combined with low inherent toxicity and high blood–brain barrier (BBB) permeability are promising therapeutics for the prevention and treatment of neurodegenerative diseases [29]. Lumbricus, the dried body of the earthworm *P. aspergillum*, has long been used in traditional East Asian medicine for several disorders including high fever, seizures in children, asthma, and high blood pressure. However, the effects and detailed molecular mechanisms of Lumbricus on neuroinflammation and nerve damage are still unknown. Therefore, that was the focus in this study, and we demonstrate that Lumbricus can indeed suppress both LPS-stimulated microglial activation and glutamate-induced neurotoxicity with no detectable inherent toxicity over a wide effective dose range. Furthermore, we identified potential active components for the development of more targeted therapeutics.

Pretreatment of BV2 microglia with LWE at non-cytotoxic concentrations inhibited LPS-induced NO release and reduced both the upregulated expression and enhanced secretion of proinflammatory cytokines (Figure 1), possibly by blocking MAPK phosphorylation and NF-κB nuclear translocation (Figure 2) as both are required for the induction of numerous inflammatory mediators [7,8]. Further, LWE suppressed LPS-induced upregulation of iNOS, consistent with the effects on NO release, and the upregulation of COX-2, suggesting additional inhibitory effects on downstream prostaglandin signaling (Figure 3). Significantly, LWE also enhanced the antioxidant capacity in BV2 cells. Heme oxygenase-1 is an antioxidant induced by nuclear translocation of Nrf-2 which forms a negative feedback loop with iNOS [9,10,11]. Thus, upregulation of HO-1 and nuclear translocation of Nrf-2 (Figure 3) may explain the observed downregulation of iNOS.

Formation of the NLRP3 inflammasome is required for glial cell activation and neuroinflammation, and excess microglial inflammasome activity is indeed attracting attention as a potential mediator of chronic neurodegeneration in disorders such as Alzheimer’s disease [30]. Formation of the NLRP3 inflammasome is inhibited by SIRT2 while P2X7 is known to activate NLRP3 [14,17]. Our Western blot analysis demonstrated that LWE can inhibit the expression of caspase-1/IL-18 and downregulate expression of P2X7 (Figure 4). Collectively, these findings suggests that LWE inhibits the inflammatory response of microglial cells to LPS by suppressing MAPK/NF-κB/NLRP3 pathways as well as by activating the HO-1/Nrf-2 pathway.

Neuroinflammation is strongly associated with accumulation of ROS and reactive nitrogen species in the brain [31], while oxidative stress can induce neuroinflammation, indicating that these pathogenic processes are mutually reinforcing and thus concomitant under most conditions. Therefore, in this study, we investigated not only the inhibitory effect of Lumbricus on neuroinflammation, but also its protective effect against neurotoxicity caused by oxidative stress mediated by HT22 hippocampal cells. Initially, we explored the anti-neuroinflammatory and neuroprotective effects of LWE and LEE, respectively. Our results show that LWE had a better inhibitory effect on neuroinflammation, and LEE had a better protective effect on neurotoxicity, so specific studies on each efficacy were conducted. LEE directly reduced glutamate-induced ROS accumulation, MDA expression, and neuronal cell death within the same dose range as LWE suppressed neuroinflammation. Moreover, similarly to LWE, LEE had no inherent cytotoxicity (Figure 5). This cytoprotection was due at least in part to suppression of glutamate-induced apoptosis as evidenced by Annexin V/PI staining and Western blotting showing reciprocal regulation of pro- and antiapoptotic proteins (Figure 7). Therefore, Lumbricus effectively inhibits glial cell-mediated neuroinflammation and significantly improves neurotoxicity caused by oxidative stress, suggesting great potential for the prevention and treatment of neurodegenerative diseases.

Finally, HPLC analysis revealed that these extracts contained uracil, hypoxanthine, uridine, xanthine, adenosine, inosine, and guanosine, consistent with previous studies [32,33]. Adenosine alone has demonstrated neuroprotective efficacy [34,35], while inosine has been reported to attenuate post-stroke neuroinflammation by modulating inflammasome-mediated microglial activation [36], reduce aging-associated oxidative stress and neuroinflammation in female rats [37], and protect against ischemic brain injury [38]. Guanosine was also found to protect the hippocampus against injury from oxygen and glucose deprivation in part by controlling inflammatory pathways [39,40]. Therefore, it appears that the anti-neuroinflammatory effects of LWE are conferred by inosine and guanosine, while the neuroprotective effects of LEE may be conferred by adenosine, inosine, and guanosine. In addition, since the content of adenosine for which neuroprotective efficacy was reported was much higher in LEE than in LWE, it may be that the relatively better neuroprotective efficacy of LEE is due to this. On the other hand, it is still unclear precisely which constituents confer the superior anti-neuroinflammatory efficacy of LWE compared to LEE. Referring to the above quantitative analysis results and previous reports demonstrating the anti-inflammatory effects of inosine and guanosine in cell models [41,42,43], it is believed that the content of the two components included in LWE may contribute to the anti-neuroinflammatory effect to some extent. Likewise, based on previous studies on the neuroprotective effects of adenosine, inosine, and guanosine [44,45,46], it is believed that the content of these components in LEE may contribute to the neuroprotective effect. Additionally, in order to have potential as a treatment for neurodegenerative diseases, BBB permeability of the material itself or its components must be prerequisite. Some previous studies have demonstrated that the components hypoxanthine, uridine, adenosine, inosine, and guanosine of Lumbricus, which we identified through HPLC analysis, exhibit BBB permeability [47,48]. However, nucleosides such as adenosine, inosine, and guanosine are substances that exist widely in many types of animals and are not specific ingredients of Lumbricus. Therefore, although the above nucleosides may contribute to some extent to the anti-neuroinflammatory and neuroprotective effects of LWE and LEE, they cannot be concluded to be bioactive components of Lumbricus extract. Research on specific bioactive components of Lumbricus has not been actively conducted before and is an area that needs to be explored in the future. Therefore, the scope of what we want to prove in this study is the anti-neuroinflammatory activity of Lumbricus extract LWE and the neuroprotective effect of LEE. The search for specific bioactive components in Lumbricus and investigation into their bioactivity should be conducted in further research.

## 5. Conclusions

LWE inhibited LPS-induced activation of BV2 microglia as evidenced by reduced expression of proinflammatory cytokines (TNF-α, IL-6, IL-1β), enzymes (iNOS, COX-2), and signaling pathways (MAPK/NF-κB/NLRP3). In addition, LWE promoted activation of the antioxidant HO-1/Nrf-2 pathway. We also found that LEE mitigated glutamate-induced oxidative stress, necrosis, and apoptosis of HT22 cells. Therefore, Lumbricus extracts or extract-based formulations may be effective treatments for neurodegenerative diseases associated with neuroinflammation, oxidative stress, and neurotoxicity.

## Figures and Tables

**Table 1:** Primers used for RT-qPCR.

Target Gene	Primer Sequence
TNF-α	F: 5′-TTCTGTCTACTGAACTTCGGGGTGATCGGTCC-3′
	R: 5′-GTATGAGATAGCAAATCGGCTGACGGTGTGGG-3′
IL-6	F: 5′-TCCAGTTGCCTTCTTGGGAC-3′
	R: 5′-GTGTAATTAAGCCTCCGACTTG-3′
IL-1β	F: 5′-ATGGCAACTGTTCCTGAACTCAACT-3′
	R: 5′-CAGGACAGGTATAGATTCTTTCCTTT-3′
iNOS	F: 5′-GGCAGCCTGTGAGACCTTTG-3′
	R: 5′-GCATTGGAAGTGAAGCGTTTC-3′
COX-2	F: 5′-TGAGTACCGCAAACGCTTCTC-3′
	R: 5′-TGGACGAGGTTTTTCCACCAG-3′
HO-1	F: 5′-TGAAGGAGGCCACCAAGGAGG-3′
	R: 5′-AGAGGTCACCCAGGTAGCGGG-3′
β-actin	F: 5′-AGAGGGAAATCGTGCGTGAC-3′
	R: 5′-CAATAGTGATGACCTGGCCGT-3′

F, forward; R, reverse.

**Table 2:** Primary and secondary antibodies used for Western blot analysis.

Antibody	Corporation	Product No.	RRID	Dilution Rate
P-ERK	Cell Signaling	#4377	AB_331775	1:1000
ERK	Cell Signaling	#9102	AB_330744	1:1000
P-p38	Cell Signaling	#9211	AB_331641	1:1000
P38	Cell Signaling	#9212	AB_330713	1:1000
P-JNK	Cell Signaling	#9251	AB_331659	1:1000
JNK	Cell Signaling	#9252	AB_2250373	1:1000
β-actin	Cell Signaling	#4970	AB_2223172	1:1000
NF-κB p65	Cell Signaling	#8242	AB_10859369	1:1000
TBP	Cell Signaling	#8515	AB_10949159	1:1000
iNOS	Cell Signaling	#2982	AB_1078202	1:1000
COX-2	Cell Signaling	#4842	AB_2084968	1:5000
HO-1	Cell Signaling	#82206	AB_2799989	1:1000
Nrf-2	Cell Signaling	#12721	AB_2715528	1:1000
NLRP3	Cell Signaling	#15101	AB_2722591	1:1000
C-Caspase-1	Cell Signaling	#89332	AB_2923067	1:1000
IL-18	Cell Signaling	#57058		1:1000
SIRT2	Cell Signaling	#12650	AB_2716762	1:1000
P2X7	Cell Signaling	#13809	AB_2798319	1:1000
BAX	Cell Signaling	#2772	AB_10695870	1:1000
Bcl-2	Cell Signaling	#3498	AB_1903907	1:1000
AIF	Cell Signaling	#4642	AB_2224542	1:1000
PARP	Cell Signaling	#9532	AB_659884	1:1000
2nd anti-mouse	Cell Signaling	#7076	AB_330924	1:5000
2nd anti-rabbit	Cell Signaling	#7074	AB_2099233	1:5000

**Table 3:** Regression data and contents of seven compounds in LWE and LEE.

	Linear Regression Equation ^a^	Correlation Coefficient (*n* = 5)	LOD ^b^ (µg/mL)	LOQ ^c^ (µg/mL)	Sample	*t_R_* (min)	Measured Amount (µg/mL)	RSD (%)	Contents (μg/mg)
1	*y* = 0.3665*x* + 0.0024	1.0000	0.08	0.24	LWE	5.63	3.77 ± 0.01	0.65	0.15
					LEE	5.63	6.71 ± 0.02	0.93	0.27
2	*y* = 0.4002*x* − 0.0423	0.9999	0.50	1.52	LWE	8.77	24.72 ± 0.01	0.06	0.99
					LEE	8.77	42.55 ± 0.07	0.39	1.70
3	*y* = 0.1998*x* − 0.0104	1.0000	0.33	1.00	LWE	10.24	27.79 ± 0.14	2.58	1.11
					LEE	10.25	57.49 ± 0.11	0.94	2.30
4	*y* = 0.9758*x* + 0.0488	0.9995	0.02	0.05	LWE	11.81	0.87 ± 0.02	2.39	0.03
					LEE	11.8	1.63 ± 0.04	2.47	0.07
5	*y* = 0.2662*x* + 0.0414	1.0000	0.01	0.01	LWE	13.39	4.31 ± 0.02	1.77	0.17
					LEE	13.39	17.67 ± 0.06	1.30	0.71
6	*y* = 0.2061*x* + 0.0073	1.0000	0.51	1.54	LWE	21.07	79.46 ± 0.03	0.18	3.18
					LEE	21.05	150.48 ± 0.26	0.83	6.02
7	*y* = 0.2612*x* + 0.0354	1.0000	0.50	1.52	LWE	22.42	18.65 ± 0.14	2.92	0.75
					LEE	22.41	33.53 ± 0.11	1.21	1.34

^a^ y: peak area; ^b^ the limit of detection; ^c^ the limit of quantification.

## Data Availability

The data are contained within the article.
